# Efficiency and Predictability of Coronal Maxillary Expansion Repercussion with the Aligners System: A Retrospective Study

**DOI:** 10.3390/dj11110258

**Published:** 2023-11-06

**Authors:** Ana Sofia Rocha, Maria Gonçalves, Ana Catarina Oliveira, Rui M. S. Azevedo, Teresa Pinho

**Affiliations:** 1 Oral Pathology and Rehabilitation Research Unit (UNIPRO)—Oral Pathology and Rehabilitation Research Unit, University Institute of Health Science (IUCS), CESPU, 4585-116 Gandra, Portugal; a27132@alunos.cespu.pt (A.S.R.); a26895@alunos.cespu.pt (A.C.O.); 2 TOXRUN—Toxicology Research Unit, University Institute of Health Sciences (IUCS), CESPU, CRL, 4585-116 Gandra, Portugal; mprazeres.goncalves@iucs.cespu.pt (M.G.);; 3 IBMC—Molecular and Cellular Biology Institute, i3S—Health Innovation and Research Institute, University of Porto, 4200-135 Porto, Portugal

**Keywords:** tooth movement, facial pattern, Invisalign, effectiveness, expansion, clear aligners

## Abstract

The Invisalign^®^ system (SmartForce^®^ G8) aims to guarantee aesthetics and provide good orthodontic treatment results. Dentoalveolar expansion is possible with clear aligners and can be used to correct dentoalveolar crossbite, resolve crowding or modify the arch shape. Despite the treatment’s effectiveness, there is still disagreement among professionals concerning its true clinical potential. This study aimed to analyze the effectiveness and predictability of coronal tooth expansion movement in permanent dentition in patients who had completed the first phase of treatment with Invisalign^®^ orthodontic aligners. Materials and Methods: The tooth movement tables of 75 previously selected cases were analyzed in terms of dental-arch width and expansion efficiency, through the Invisalign^®^ platform, considering the pre-treatment (T0), planned treatment (TP) and post-treatment models (T1) using ClinCheck Pro^®^ 6.0 software. All patients were treated by an orthodontic specialist and Invisalign^®^ Diamond Provider in a private practice (T.P.). Results: Difference between T1 and T0: for each maxillary and mandibular measurement, there was a statistically significant difference between pre- and post-aligner treatment values. The greatest amount of expansion occurred in both the upper and the lower premolars. Difference between TP and T1: for each maxillary measurement, statistically significant differences were verified for the molar and canine. At the mandibular level, statistically significant differences were only verified in the first molar. Conclusions: The Invisalign^®^ clear aligners are effective for simultaneous intra-arch expansion in both jaws.

## 1. Introduction

The growing concern of orthodontic patients about the aesthetic impact of their treatments has driven the introduction of new appliances which fulfill this aesthetic demand. In 1946, Harold Kesling originally had the idea to use transparent, thermosetting plastic devices for orthodontic purposes and, several decades later, Align Technology^®^ was the first company to produce clear aligners (CA). The key benefits of the use of CA lie in their comfort and aesthetics, in the reduction of in-office time, and in the ease of cleaning compared to conventional fixed appliances. Arch expansion may be required to widen the dental arches as a means of creating space for crowding correction or enhancing the appearance of smiles. It can also correct dentoalveolar posterior crossbites [1,2,3,4,5]. To reduce the risk of gingival recurrence and recession, several studies which evaluated the expansion of dental arches have recommended that the expansion limit of the arch width should be a maximum of 2–3 mm per quadrant [6].

There are two different types of expansion: dentoalveolar and orthopedic. When transverse deficits and/or crowding are mild, dentoalveolar expansion is an option for treatment. It improves the transverse dimension of the smile and corrects posterior crossbites whenever they are of dentoalveolar origin.

However, orthopedic expansion procedures are used when maxillary compression is moderate to severe and there is bone-base involvement [7].

As previously reported in the literature, clear-aligner therapy is usually performed in combination with other orthodontic auxiliaries to facilitate tooth movement and increase movement predictability. These orthodontic auxiliaries can include attachments, interarch elastics, and interproximal reduction. Beyond virtual planning, the mechanical qualities of the thermoplastic materials used in the aligners and the design of the attachments, which can vary in size, shape, and position based on the orthodontist’s choice, are both directly related to the effectiveness of tooth movement [3,8].

Some tooth movements, such as extrusion and rotation, have proven to be less predictable and have required extra attention, especially for certain teeth. This in turn has led to the evolution of the system. Optimized designs and better material construction have allowed aligners to produce a greater range of motion in a shorter treatment time. The strategic arrangement of attachments increases the delivery of force and, consequently, favors tooth movement. Furthermore, the new SmartTrack^®^ material (LD30) has several advantages over the old material (Ex30), including a more precise and comfortable aligner fit, a higher degree of elasticity, and chemical stability [9,10,11,12,13]. The orthodontist’s role is to achieve occlusal and facial results that can provide the greatest benefit to the individual. The limitations of orthodontic treatment are largely determined by soft tissues, from the point of view of function and stability, as well as aesthetics. It is also known that the shape of the tooth arch is related to the vertical dimension, and that the jaw’s transverse dimension is related to vertical skeletal growth. The orthodontist must plan treatment within the limits of the soft tissues’ adaptation and contours, taking into account the patient’s facial biotype as a reference [14,15].

Despite agreement as to the treatment’s efficacy, there is still disagreement among professionals about its true clinical potential [4,5,8,16,17]. Therefore, the purpose of this clinical study was to investigate the effectiveness and predictability of upper- and lower-arch expansion using Invisalign^®^ aligners as an orthodontic appliance, with the aim of verifying whether there were significant differences before and after the first stage of treatment. 

## 2. Materials and Methods 

### 2.1. Study Design

This research sought to examine the degree of expansion resulting from the first stage of orthodontic treatment with clear aligners. Therefore, two evaluations were performed at different times: one before the start of treatment and another after the first series of aligners. This study also aimed to analyze the impact of one of the cephalometric characteristics of facial biotype. Considering the objectives outlined for this investigation and the time and resources available, a quantitative, comparative, and observational longitudinal cohort study design was chosen.

### 2.2. Samples and Eligibility Criteria

This study focused on patients with complete permanent dentition (excluding third molars) who were undergoing orthodontic treatment with Invisalign^®^ clear aligners in a private clinic in the northern region of Portugal, by a specialist in orthodontics and Invisalign^®^ Diamond Provider (T.P.). Only patients whose treatment started in 2020, after the appearance of SmartForce^®^ G8, were selected. This way, only aligners made with SmartTrack^TM^ material were used in the present study. A population with permanent dentition and a planned expansion of ≥2 mm in at least one interdental width was chosen as the sample for this study, in order to avoid normal transverse increases in jaw growth, which would have affected our results. The patients were told to wear each aligner for as long as possible (20–22 h each day), only taking them out to eat and perform oral hygiene. The aligners were changed every 7 or 10 days, as recommended by Invisalign^®^ aligner protocols. The patients’ compliance was verbally confirmed at each appointment. The sample selection criteria are shown in Table 1.

### 2.3. Ethical Principles

This research is part of a project that was approved by the Ethics Committee of the University Institute of Health Sciences (CESPU), with reference 4/CE-IUCS/2023.

### 2.4. Data Collection Procedures

**Figure 1:**
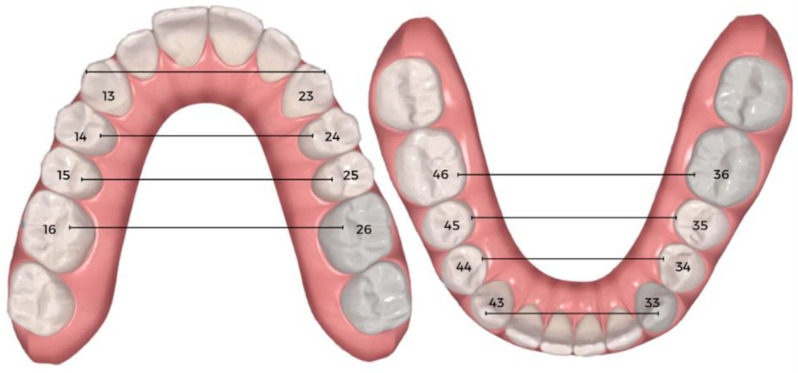
Example of interdental width linear measurement using Clincheck^®^ software (T1 stage).

The initial sample consisted of one hundred and five participants treated with the Invisalign^®^ SmartTrack aligner. Subsequently, in accordance with the selection criteria, the sample was reduced to seventy-five participants. Their records were reviewed. By analyzing the clinical records, data were collected regarding gender and age at the commencement of orthodontic treatment with clear aligners. The treatment protocols used to correct transverse discrepancy in these participants did not include tooth extractions or the use of auxiliaries other than attachments. All patients were treated with clear aligners and their expansion planning generally required expansion with zero torque. In the case of the present study, none of the patients used crossbite elastics. The 3D digital models were obtained through the intra-oral scanner before treatment (T0) and after treatment with the first series of aligners (T1), using Itero^®^ software version 1.34.0.3, and evaluated using Clincheck Pro^®^ 6.0 software. The tooth movement tables on the ClinCheck^®^ treatment-planning software were consulted for dental-arch width and expansion efficiency, considering the pre-treatment (T0), planned treatment (TP), and post-treatment (T1) models provided by the ClinCheck^®^ software. Linear measurements of the interdental width at stages (T0) and (T1) were recorded, including the intercanine width between the cusp tips, the interpremolar widths between the palatal cusp tips of the first and second premolars, and the intermolar width between the tips of the mesopalatine cusps of the first molars (Figure 1). This way the expansion studied would be purely dental.

Cephalometric images were collected from all participants in the sample and cephalometric tracing was performed. Through the cephalometric tracing, it was possible to determine overjet, overbite, and, with the intersection of the Frankfurt plane with the mandibular plane, the FMA values. An FMA of 25 ± 5 degrees is within the normal range and is associated with normodivergent patients. A high-angle patient, otherwise known as a hyperdivergent patient, has an FMA of 30 degrees or more. A low-angle patient, or hypodivergent patient, has an FMA of 20 degrees or less [18].

### 2.5. Hypotheses to Be Tested 

The hypotheses to be tested in the population included in this study were:

I. H0: Expansion does not differ significantly between the beginning and end of the first stage of orthodontic treatment with clear aligners.
II. H0: Expansion does not differ significantly between what is planned by the orthodontist and what is obtained at the end of the first stage of orthodontic treatment with clear aligners.
III. H0: The results of expansion planning at the end of the first stage of orthodontic treatment with clear aligners are not influenced by the facial biotype.

### 2.6. Statistical Analysis

Data analysis was performed using the IBM^®^ SPSS^®^ software (Statistical Program for Social Sciences), version 29.0 for Windows. Descriptive statistics were produced which provided estimates for frequencies and percentages, means, medians, standard deviation, minimums and maximums. The Shapiro–Wilk test was used to assess sample normality, with no evidence for rejecting the null hypotheses. The normality of the data led us to adopt the dependent *t*-test to compare maxillary and mandibular expansion before and after placement of the first set of aligners. To measure the magnitude of the effect, Cohen’s d was used with the following guidelines: |d| ≤ 0.20 expected as a small effect, |d| = 0.50 expected as a moderate effect, and |d| ≥ 0.80 expected as a large effect [19]. To compare maxillary and mandibular expansion before and after placement of the first set of aligners according to facial biotype, one-way ANOVA was used, followed by the Bonferroni test. Effect sizes for ANOVA were determined using η^2^ values, with the thresholds considered to be η^2^ = 0.01 for a small effect, η^2^ = 0.06 for a medium effect, and η^2^ = 0.14 for a large effect. The significance level was set at 0.05.

## 3. Results 

### 3.1. Characteristics of the Clinical Study Sample

The first sample consisted of 105 participants. After the first stage of treatment with aligners had been performed, a second selection process was carried out. Subsequently, in accordance with the selection criteria, a further 30 individuals were eliminated, the majority as a result of not having a planned expansion of ≥2 mm in at least one interdental width.

**Figure 2:**
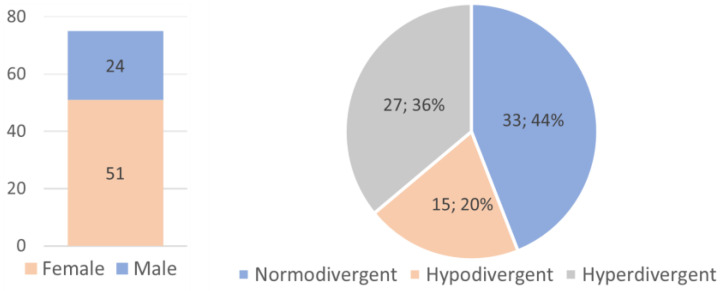
Sample distribution according to gender and facial biotype.

In line with the inclusion and exclusion criteria, the final sample consisted of 75 individuals aged between 11 and 49 years old (mean = 23.30; SD = 9.81), of which 51 (68.0%) were female and 24 (32.0%) were male. In terms of facial biotype, 33 (44.0%) were normodivergent, 27 (36.0%) hyperdivergent, and 15 (20.0%) hypodivergent (Figure 2). The clinical study of the sample revealed that the majority had normal overbite (78.7%; n = 59) and normal overjet (74.7%; n = 56). With regards to Molar Class, it was found that 56.0% of individuals had Left Molar Class I and 45.3% Right Molar Class I. As for the attachments, the majority of the sample (93.3%) used optimized and conventional rectangular attachments, with conventional rectangular attachments located mainly on molars and optimized attachments on premolars and canines (SmartForce^®^ G8).

Of the total sample, 10 patients had crossbites in the analyzed areas. Four patients had a unilateral crossbite in the premolar area, one had a unilateral crossbite in the canine area, three had a bilateral crossbite in the first molar area, one had a bilateral crossbite in the canine area, and one had a unilateral crossbite from the premolar up to the first molar.

### 3.2. Analysis of the Efficacy of Maxillary and Mandibular Expansion

**Figure 3:**
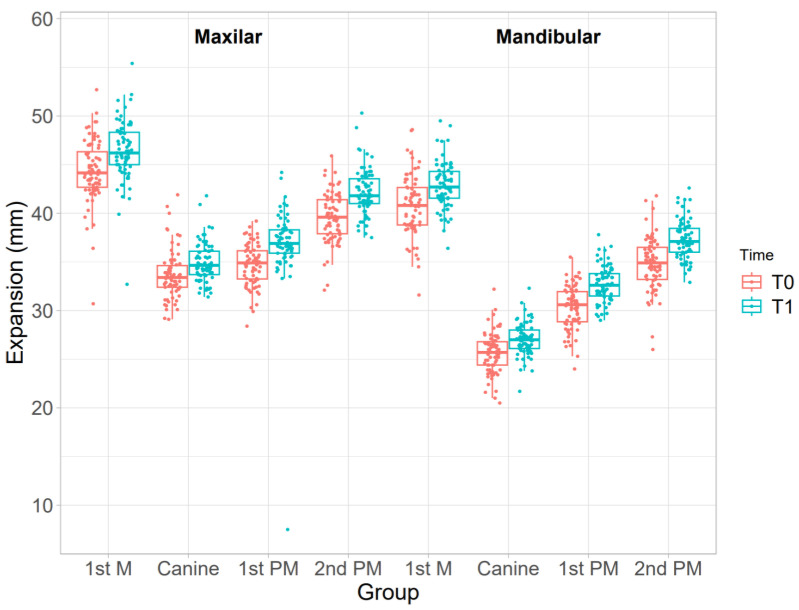
Boxplots showing the differences in expansion movement between pre-treatment and post-treatment in the upper and lower arch (T1-T0).

**Figure 4:**
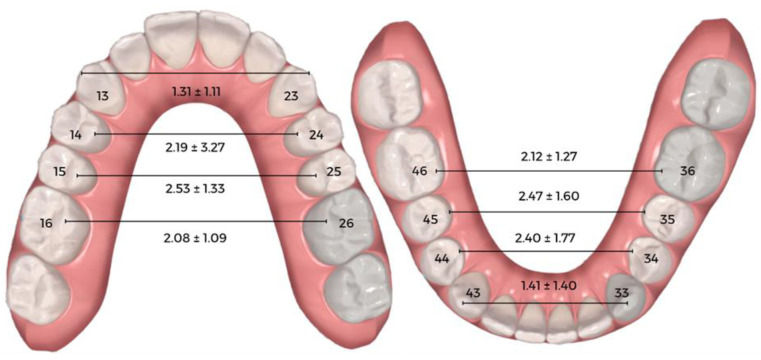
Efficacy outcomes expressed in millimeters.

Table 2 and Figure 3 summarize the differences between maxillary and mandibular expansion before aligner placement and after the first set of aligners. For each maxillary measurement, there was a statistically significant difference between pre- and post-aligner values. The greatest increase in maxillary width was detected in the first and second premolars, with expansions of 2.19 ± 3.27 mm and 2.53 ± 1.33 mm, respectively. For each mandibular measurement, there was also a statistically significant difference at the level of the whole arch. The greatest increase in mandibular width was detected in the first and second premolars, with expansions of 2.40 ± 1.77 mm and 2.47 ± 1.60 mm, respectively. For ease of interpretation, these results are illustrated in Figure 4.

### 3.3. Predictability Analysis

**Figure 5:**
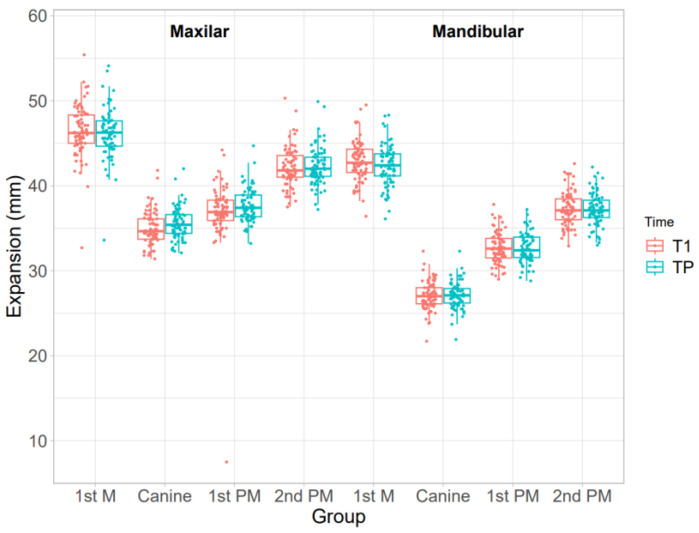
Boxplot showing the differences in expansion movement between actual post-treatment and initially predicted ClinCheck^®^ expansion.

The predictability/efficiency of the Invisalign^®^ ClinCheck software was determined after the completion of treatment with the first series of aligners (Table 3 and Figure 5) by comparing the expansion achieved on the digital models (T1) and the planned expansion (TP). At the maxillary level, there were statistically significant differences between the planned and achieved expansions. This occurred at the first molar level (*p* = 0.036), where the achieved expansion was 0.23 ± 0.94 mm greater than expected, and at the canine level (*p* < 0.001), where the achieved expansion was 0.56 ± 0.64 mm less than predicted. The expansion achieved of the first and second premolars was below the expansion expected. At the mandibular level, statistically significant differences were only verified in the first molar (*p* < 0.001), and the expansion obtained was 0.54 ± 0.85 mm greater than that predicted by the ClinCheck^®^ software. As regards the canine, the expansion obtained was 0.06 ± 0.43 lower than the predicted value, while in the first and second molars the expansions obtained were, respectively, 0.04 ± 0.84 mm and 0.08 ± 0.72 mm higher than the expansion planned using the ClinCheck^®^ software. In percentage terms, predictability at the maxillary level was 113% at the first molar level, 70.1% at the canine level, 54.75% at the first premolar level, and 94.8% at the second premolar level. 

**Figure 6:**
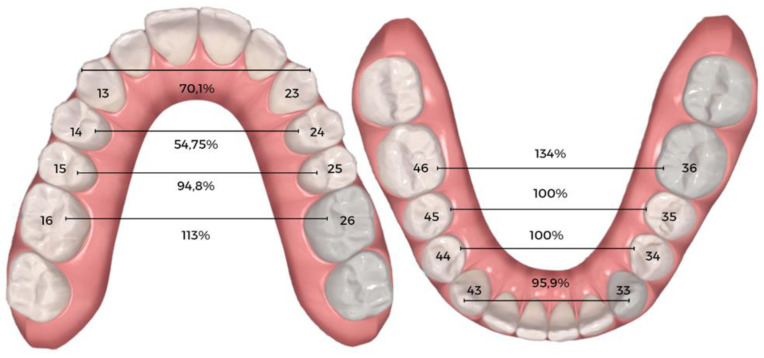
Predictability outcomes expressed in percentage.

At the mandibular level, predictability was 134% at the first molar level, 95.9% at the canine level, and 100% at the first premolar and second premolar levels, respectively (Figure 6).

When comparing the mean expansion required (Planned T0—Measure T0) at the maxillary and mandibular level before the start of treatment with the effectiveness achieved after placement of the aligners (Planned T1—Measure T1), statistically significant differences were found in all of the measurements, with the need for expansion dropping significantly in all of them (Table 4).

### 3.4. Mandibular and Maxillary Expansion According to Facial Biotype

When comparing maxillary expansion before placement of aligners and after the first series of aligners according to facial biotype, it was found that for all maxillary measurements, both before and after the placement of aligners, hypodivergent individuals presented higher mean values than normodivergent and hyperdivergent participants. However, these differences were not statistically significant (Table 5). With regards to mandibular expansion, both before and after the placement of aligners, hypodivergent individuals also presented higher mean values in all mandibular measurements, compared to the other biotypes. These differences reached statistical significance in the first molar, before the placement of aligners, (F (2.72) = 3.50; *p* = 0.035), and also after the treatment (F (2.72) = 3.32; *p* = 0.042). The major causes of these results stem from the differences between hypodivergent (42.20 ± 2.77) and normodivergent (39.82 ± 3.26) individuals (*p* = 0.04) in the first case, as well as at the end of the first series of aligners, with hypodivergent individuals presenting mean values of expansion (44.05 ± 1.79) that are significantly higher than those of normodivergent individuals (42.22 ± 2.53) (*p* = 0.04). Curiously, at the level of the first premolar, differences between hypodivergent (33.71 ± 1.79) and normodivergent (32.37 ± 1.75) (*p* = 0.049) groups were found, but only after the treatment.

## 4. Discussion

### 4.1. Effectiveness and Predictability of the Invisalign System

In the present study, a population of participants aged 11 years and older was selected, due to the inclusion criterion of requiring complete dentition. Chronologically, jaw growth ends following a defined sequence in three planes. Transverse growth of the mandible or maxilla ends first, followed by growth in length and height, and the jaw generally reaches its definitive size before the onset of puberty. Changes related to growth during adolescence do not have an effect, or have a minimal effect, on the width of the dental arches [20]. Indeed, some more recent studies demonstrate that growth continues beyond 11 years of age; however, these are not significant values. For example, the upper intermolar width measured at the cusp tips increases in males at a rate of 0.47 mm/year and in females at a rate of 0.66 mm/year. In addition, the lower intermolar width increases in males at a rate of 0.42 mm/year and in females at a rate of 0.70 mm/year. Furthermore, it is agreed that the increase in intermolar width ceases with the development of complete permanent dentition [21,22]. Another criterion used in our study was that individuals should have a planned expansion of ≥2 mm in at least one interdental width (Table 1). Through this, and by adding the criterion of excluding individuals with deciduous or mixed dentition, as expressed in the selection criteria for participants in the present study, we avoided the presence of significant transversal growth in our sample, and therefore reduced the likelihood that the expansion found was due to natural bone growth. 

In response to the first hypothesis of whether the Invisalign^®^ system is capable of effectively producing expansion movement, we discovered that Invisalign^®^ clear aligners are a successful method for achieving transverse expansion, as the results obtained showed an increase in all dental widths to a greater or lesser extent. These results are consistent with other previously published studies [4,16,23,24,25]. As the results show, all widths underwent significant increases as a result of the treatment, with the greatest amount of expansion occurring in both the upper and lower premolars. Some authors obtained identical results and argued that this may be due to the positioning of the premolars in a straight line, resulting in a greater tendency towards expansion [16,24,26,27]. This study did not distinguish between expansion due to tooth inclination and expansion due to movement in the body; however, some studies claim that expansion is possible with Invisalign aligners, although mainly through tilting movements [3,16,25]. A recent study showed that ClinCheck^®^ predicts more body movement than the Invisalign system is capable of achieving [28]. 

The second main objective of our review was to assess the predictability of measurements planned using ClinCheck^®^ software at the end of the first treatment stage with CA. Almost all differences between ClinCheck^®^ planning and clinical outcome were not statistically significant. This shows that the software was able accurately to predict the amount of change that took place. Most previous studies are in line with our results [4,17,24,29]. The exceptions reside in the lower first molar and upper first molar, where the expansion achieved was greater than that predicted, and in the upper canine, where the expansion achieved was lower than that predicted. The data obtained indicate that, in general, molars expanded more than planned, while canines expanded less than predicted by the ClinCheck^®^ software. 

The lower canines and premolars were the teeth for which the values predicted by the software were closest to the achieved values. In absolute terms, the results show that treatment in the lower arch achieved smaller differences between planned and achieved values than treatment in the upper arch. The fact that the amount of change requested in the lower arch is typically lower than that in the upper arch may help to explain this. Additionally, resistance is decreased due to the fact that the upper arch is being expanded multiple times simultaneously [17].

Based on our results, we can see that there was overexpansion with statistically significant values at the level of the upper and lower first molars of 0.23 ± 0.94 mm and 0.54 ± 0.85 mm, respectively. The mechanism by which this overexpansion may occur is not intentionally created by planned body expansion, but is instead unintentionally manifested as tipping. This results in greater movement of cusp tips than programmed. In fact, tipping and torque are two of the most difficult movements to control using this system [30]. This is in line with earlier research, which discovered a tendency for planned body movement to be expressed through torque and tipping. Other possible explanations for overexpansion could include software discrepancies, the lower level of force exerted at the end of the aligner action due to greater elasticity and distortion in this area, rotation around the palatal root of the hinged tooth during expansion, or some degree of inbuilt overcorrection engineered into Invisalign’s aligner fabrication system [17,24,30,31]. However, further studies are needed to confirm these hypotheses. It must be emphasized that these results represent only the outcome after the first set of Invisalign^®^ aligners in a group of patients in which an increase of 2 mm or more was planned in at least one interdental measurement. We must bear in mind that, in the present study, the expansion is purely dental, as there is no bone reference point evaluated. 

### 4.2. Facial Patterns

Facial analysis is performed frequently and unconsciously by everyone and directly influences our perception of the people we interact with. This highlights the importance of taking facial appearance into consideration during diagnosis and orthodontic planning, meaning that, in addition to obtaining good occlusal results, the orthodontist must aim to maintain or create harmony between the face and teeth [32]. Our results showed that the teeth of hypodivergent individuals had a greater interdental width than those of normodivergent and hyperdivergent individuals, both at T0 and at T1. This is to be expected because, when the face is wider, as is the case with hypodivergent patients, the arches and the interdental widths are naturally wider at the beginning of the treatment. Conversely, if the arches are compressed, transversal expansion will probably be planned to maintain the correspondence between the facial biotype and the shape of the dental arch and, as such, greater interdental widths are also observed at the end of the first phase of treatment. This finding is true for all maxillary and mandibular measurements, but the results for the maxilla are not considered statistically significant. However, for the mandibular measurements, there are statistically significant differences at the level of the first premolar and first molar among normodivergent and hypodivergent individuals at the end of the first phase of treatment. Since, in general, the differences between the three facial biotypes do not reach statistically significant values, we do not reject the null hypothesis. To the best of our knowledge, this is the first study to verify whether facial biotype influences treatment outcome in arch expansion. Thus, we have no means of comparison with previous studies.

### 4.3. Clinical Relevance

It has been reported that a high percentage of patients treated with Invisalign^®^ aligners (70% to 80%) could need a refinement or additional aligners (AA). This suggests that ClinCheck^®^’s treatment planning accuracy is poor, and this may be a result of the practitioner’s inexperience with the technique, flaws in the software, or a lack of patient compliance [3]. Because in this study we only evaluated alterations resulting from the first phase of treatment, the need for AA was not verified, but, based on the results, we believe that it is necessary to make adjustments throughout the treatment [33]. Despite the high level of accuracy found in this study, there was a statistical difference between the predicted ClinCheck^®^ plan and the expansion achieved for some measurements. The literature suggests that planning overexpansion in the software could be an alternative that would help to improve the treatment result. This could be especially productive in the case of the canines, which in this study proved to be the teeth with the least expansion, with the maxillary canine failing to reach planned values with statistically significant levels. Aids such as crossbite elastics can be utilized to enhance the teeth’s transverse relationship [17]. The control of tooth inclination is a different consideration that the orthodontist may want to address in treatment planning. In previous studies, it was found that teeth are more easily inclined than physically expanded, especially in the case of canines and first molars. Although we have not studied it, we can assume that this was the cause of the overexpansion observed in the upper and lower molars, but further studies will be needed to confirm this hypothesis [23].

### 4.4. Limitations of the Study

This study was designed to evaluate the effectiveness of the Invisalign^®^ system in achieving the expansion planned by the orthodontist for the first phase of treatment only, using the ClinCheck^®^ software. However, the stability of the changes after this was not assessed. Another limitation of the study was the non-evaluation of the second molars: the software in question does not provide us with data for this group of teeth. The selection of participants who finished the first series of aligners without misfits could be considered a selection bias, but on the other hand it could be useful to gauge the highest level of expansion the professional can achieve without aligner misfits.

Since the Invisalign^®^ system evolves quickly, there may be some discrepancies in the comparison of the results of the present study with others that used different versions of the ClinCheck^®^ software and different techniques, namely in the use of attachments. Additionally, in order to reduce the variability of test results, and standardize the process of measuring interdental widths, the standardization criteria used in the recruitment and selection of candidates must be objective, repeatable, and described in future studies. It would be interesting to carry out a similar study with CBCT to verify whether different types of expansion, namely expansion by body movement and expansion by tipping, influence the accuracy of the ClinCheck^®^ software. However, it is not ethically plausible to use unnecessary radiation to evaluate this parameter. It would be interesting to perform a follow-up investigation to gauge the long-term stability of the changes achieved. We also recommend that future research examines tooth movements in all dentitions in order to identify the causes of underexpansion and overexpansion and to avoid overcorrection.

## 5. Conclusions

The results of the study allowed us to conclude that clear aligners are effective in producing simultaneous intra-arch expansion in both jaws, with expansion being most efficient in the premolar area and least efficient in the canine and first molar areas. For expansion movement, predictability was very reasonable, with slight inaccuracies resulting in both underexpansion and overexpansion. In terms of the relationship between expansion and facial biotype, we conclude that hypodivergent individuals have the greatest interdental widths both at T0 and T1. In addition, hypodivergent individuals showed a greater difference in expansion movement between T0 and T1. However, further investigations are needed to understand better the mechanisms that influence expansion movement and its long-term stability.

## Figures and Tables

**Table 1:** Inclusion and exclusion criteria for the study.

**Inclusion Conditions**
Individuals with permanent dentition undergoing orthodontic treatment with clear aligners (CA);
Individuals who had already completed the first series of orthodontic treatment with CA, with no misfits;
Individuals with a planned expansion of ≥2 mm in at least one interdental width.
**Exclusion Conditions**
Individuals without complete permanent dentition up to the 2nd molars;
Individuals with a need for orthognathic surgical treatment;
Individuals with cognitive or neurological disorders, identified syndromes, history of trauma and/or tumors in the head and neck, and/or metabolic diseases that affect the joints and/or muscles;
Individuals who were being treated with anti-inflammatories, analgesics, or psychiatric medication.

**Table 2:** Descriptive statistics and statistical comparisons of pre-treatment and post-treatment changes in the upper and lower arch (T1-T0) (dependent *t*-test).

		Mean ± s.d.	T1-T0 (mm)	Mean Expansion Planned	CI 95%	*p*-Value	Cohen’s d
Maxilar							
1st M	T0	44.34 ± 3.22	2.08 ± 1.09	2.98	[1.83–2.32]	<0.001	1.09
	T1	46.41 ± 3.10					
Canine	T0	33.65 ± 2.50	1.31 ± 1.11	1.87	[1.05–1.57]	<0.001	1.12
	T1	34.96 ± 1.98					
1st PM	T0	34.67 ± 2.11	2.19 ± 3.27	2.67	[1.44–2.44]	<0.001	3.27
	T1	36.86 ± 4.02					
2nd PM	T0	39.61 ± 2.55	2.53 ± 1.33	1.84	[2.23–2.84]	<0.001	1.33
	T1	42.15 ± 2.34					
Mandibular							
1st M	T0	40.76 ± 3.11	2.12 ± 1.27	2.36	[1.83–2.41]	<0.001	1.27
	T1	42.87 ± 2.39					
Canine	T0	25.62 ± 2.08	1.41 ± 1.40	1.46	[1.09–1.73]	<0.001	1.40
	T1	27.04 ± 1.65					
1st PM	T0	30.28 ± 2.24	2.40 ± 1.77	2.39	[2.00–2.81]	<0.001	1.74
	T1	32.69 ± 1.81					
2nd PM	T0	34.79 ± 2.73	2.47 ± 1.60	1.58	[2.11–2.84]	<0.001	1.61
	T1	37.27 ± 1.97					

**Table 3:** Descriptive statistics and statistical comparisons between actual aftercare and initially predicted ClinCheck^®^ expansion (dependent *t*-test).

		Mean ± s.d.	T1-TP (mm)	CI 95%	%	*p*-Value	Cohen’s d
Maxilar							
1st M	TP	46.18 ± 3.10	0.23 ± 0.94	[0.02; 0.45]	113%	0.036	0.94
	T1	46.41 ± 2.90					
Canine	TP	35.52 ± 1.84	−0.56 ± 0.64	[−0.71; −0.41]	70.1%	<0.001	0.63
	T1	34.96 ± 1.97					
1st PM	TP	37.65 ± 1.89	−0.78 ± 3.57	[−1.61; 0.04]	54.75%	0.061	0.22
	T1	36.86 ± 4.02					
2nd PM	TP	42.28 ± 2.19	−0.13 ± 0.81	[−0.32; −0.05]	94.8%	0.160	0.81
	T1	42.15 ± 2.34					
Mandibular							
1st M	TP	42.34 ± 2.35	0.54 ± 0.85	[0.34; 0.73]	134%	<0.001	0.85
	T1	42.87 ± 2.39					
Canine	TP	27.09 ± 1.55	−0.06 ± 0.43	[−0.16; 0.04]	95.9%	0.267	0.43
	T1	27.04 ± 1.65					
1st PM	TP	32.64 ± 1.70	0.04 ± 0.84	[−0.15; 0.24]	100%	0.653	0.84
	T1	32.68 ± 1.81					
2nd PM	TP	37.19 ± 1.93	0.08 ± 0.72	[−0.09; 0.024]	100%	0.345	0.72
	T1	37.27 ± 1.97					

**Table 4:** Descriptive statistics and statistical comparisons between the mean expansion required and the effectiveness achieved after placement of the aligners.

		Mean ± s.d.	Differences	CI 95%	*p*-Value	Cohen’s d
Maxilar						
1st M	Align T0-T0	1.86 ± 1.34	−1.31 ± 1.25	[−1.60; −1.03]	<0.001	1.25
	Align T1-T1	0.54 ± 0.56				
Canine	Align T0-T0	1.98 ± 1.17	−1.44 ± 1.09	[−1.69; −1.18]	<0.001	1.09
	Align T1-T1	0.55 ± 0.54				
1st PM	Align T0-T0	2.99 ± 1.44	−2.11 ± 3.24	[−2.80; −1.32]	<0.001	3.22
	Align T1-T1	0.93 ± 3.51				
2nd PM	Align T0-T0	2.68 ± 1.48	−2.14 ± 1.33	[−2.44; −1.83]	<0.001	1.33
	Align T1-T1	0.54 ± 0.55				
Mandibular						
1st M	Align T0-T0	1.97 ± 1.09	−1.55 ± 1.04	[−1.79; −1.31]	<0.001	1.04
	Align T1-T1	0.43 ± 0.44				
Canine	Align T0-T0	1.69 ± 1.22	−1.33 ± 1.22	[−1.61; −1.04]	<0.001	1.22
	Align T1-T1	0.36 ± 0.39				
1st PM	Align T0-T0	2.40 ± 1.61	−1.93 ± 1.62	[−2.30; −1.56]	<0.001	1.62
	Align T1-T1	0.47 ± 0.45				
2nd PM	Align T0-T0	2.55 ± 1.61	−2.00 ± 1.66	[−2.38; −1.62]	<0.001	1.66
	Align T1-T1	0.55 ± 0.48				

**Table 5:** Mandibular and maxillary expansion according to facial biotype.

	T0					T1				
	Normodivergent	Hyperdivergent	Hypodivergent			Normodivergent	Hyperdivergent	Hypodivergent		
	Mean ± s.d.	Mean ± s.d.	Mean ± s.d.	*p*	Effect Size	Mean ± s.d.	Mean ± s.d.	Mean ± s.d.	*p*	Effect Size
Maxilar										
1st M	44.15 ± 3.10	44.05 ± 3.84	45.29 ± 2.03	0.447	0.0–0.11	46.11 ± 2.64	45.93 ± 3.78	47.97 ± 2.17	0.09	0.0–0.18
Canine	33.89 ± 1.91	33.04 ± 2.74	34.18 ± 3.10	0.288	0.0–0.13	34.93 ± 1.49	34.64 ± 2.10	35.58 ± 2.64	0.339	0.0–0.12
1st PM	34.60 ± 2.12	34.54 ± 2.22	35.08 ± 1.96	0.706	0.0–0.07	36.10 ± 5.38	36.93 ± 2.09	38.37 ± 2.71	0.198	0.0–0.15
2nd PM	39.38 ± 2.61	39.72 ± 2.53	39.95 ± 2.54	0.750	0.0–0.07	41.78 ± 2.05	41.95 ± 2.46	43.31 ± 2.51	0.093	0.0–0.18
Mandibular										
1st M	39.82 ± 3.26 *	41.10 ± 2.80	42.20 ± 2.77 *	0.035	0.0–0.21	42.22 ± 2.53 **	43.03 ± 1.03	44.05 ± 1.79 **	0.042	0.0–0.21
Canine	25.44 ± 1.79	25.80 ± 2.10	25.60 ± 2.08	0.807	0.0–0.06	27.14 ± 1.37	26.67 ± 1.70	27.45 ± 2.05	0.308	0.0–0.13
1st PM	29.93 ± 2.32	30.48 ± 2.30	30.69 ± 1.93	0.471	0.0–0.10	32.37 ± 1.75 ***	32.50 ± 1.76	33.71 ± 1.79 ***	0.045	0.0–0.20
2nd PM	34.29 ± 2.91	35.34 ± 2.76	34.91 ± 2.20	0.336	0.0–0.12	36.75 ± 1.86	37.36 ± 1.88	38.22 ± 2.11	0.052	0.0–0.20

* Statistically significant differences in terms of 1st molar expansion at T0 between normodivergent and hypodivergent individuals (*p* = 0.04); ** Statistically significant differences in terms of 1st molar expansion at T1 between normodivergent and hypodivergent individuals (*p* = 0.04); *** Statistically significant differences in terms of 1st premolar expansion at T1 between normodivergent and hypodivergent individuals (*p* = 0.049).

## Data Availability

Data that support this study’s findings are available from the corresponding author upon request.
